# Homeostatic model of human thermoregulation with bi-stability

**DOI:** 10.1038/s41598-021-96280-0

**Published:** 2021-08-30

**Authors:** Veronika Hajnová, Filip Zlámal, Peter Lenárt, Julie Bienertova-Vasku

**Affiliations:** 1 Department of Mathematics and Statistics, Faculty of Science, Masaryk University, Brno, Czech Republic; 2 RECETOX, Faculty of Science, Masaryk University, Brno, Czech Republic

**Keywords:** Physiology, Mathematics and computing

## Abstract

All homoiothermic organisms are capable of maintaining a stable body temperature using various negative feedback mechanisms. However, current models cannot satisfactorily describe the thermal adaptation of homoiothermic living systems in a physiologically meaningful way. Previously, we introduced stress entropic load, a novel variable designed to quantify adaptation costs, i.e. the stress of the organism, using a thermodynamic approach. In this study, we use stress entropic load as a starting point for the construction of a novel dynamical model of human thermoregulation. This model exhibits bi-stable mechanisms, a physiologically plausible features which has thus far not been demonstrated using a mathematical model. This finding allows us to predict critical points at which a living system, in this case a human body, may proceed towards two stabilities, only one of which is compatible with being alive. In the future, this may allow us to quantify not only the direction but rather the extent of therapeutic intervention in critical care patients.

The discovery of homeostasis, the ongoing maintenance and defense of vital physiological variables such as blood pressure and blood sugar, is generally attributed to Walter Cannon, considered to be the primary author of this prominent principle underlying physiological regulation^1^. However, already in 1878, the French physiologist Claude Bernard described the processes of physiological control as the *milieu interieur* ^2^. The evolution of the term “homeostasis” has a fascinating history, and generally covers a large array of physiological and behavioral responses which are elicited in a precisely coordinated way to facilitate the maximum adaptiveness of the living system to the surrounding environment. Although the term homeostasis was originally used to refer to processes within living organisms, it is currently widely used to describe autonomous control in any system and is generally understood as a way of maintaining the adaptiveness of any system, either a living one or a machine.

The core principle of the hypothesis of the homeostatic regulation of a biological parameter is that all responses act together in a highly coordinated fashion to adjust bodily parameters crucial for the survival and reproduction of the living system. This means that the homeostatic regulation is precise and targeted and requires constant fine-tuning, including multiple negative feedback mechanisms, as expressed by Walter Cannon in 1945^3^.

Over the last decades, a large number of physiologically plausible models^4,5^ have attempted to conceptualize homeostatic mechanisms using a systemic approach based on regulated variables, their set points, errors, and comparative information and their proxies. This is an extremely problematic approach, as there are countless physiological parameters that characterize the living organism at a given moment, and these countless parameters can further involve countless synergies or antagonisms. One may even argue that such an approach, based on atomizing the environmental influences in various categories and evaluating the influences of these drivers in different individuals, is reductionist to the point of no longer being useful. Therefore, while many explanations on the interaction of living organisms with the environment have been proposed, starting with research of Hans Selye^6,7^, no completely satisfactory theory describing the interaction between organisms and the surrounding environment is currently available. Furthermore, no presently available methods feature a predictive function capable of making predictions about the thermal homeostasis of the body in given ambient temperatures with satisfactory precision.

In our previous study, we introduced stress entropic load (SEL), a novel variable designed to facilitate the objective physical measurement of the stress load of a living (human) body by monitoring energy and matter flows in the body using the proxy of entropy production^8^. Mathematically, SEL is a time-dependent function which may be calculated from different types of heat, temperature changes and gases exchange. In line with our initial definition, we further described an equation calculating approximate SEL for a short measurement interval. In this article, we present a novel approach to measure SEL development in humans, and we further test this approach on healthy male volunteers subjected to prolonged mental effort^9^. As the calculation itself is based on commonly measured variables, e.g. volumes of inspired/expired O2/CO2 and measurements of core/surface/ambient temperatures, the method is potentially usable in a practical setting, including under clinical conditions.

Our current goal is to link the concept of SEL with a model of human thermoregulation. Previous studies proposed several models which can be used to study mammal thermoregulation ^4,10–12^. However, while simple phenomenological models^4^ do not include all of the necessary variables for computing SEL, and thus cannot be used to model stress response during thermoregulation, complex simulation models ^10–12^ do include all of the necessary variables for computing SEL, but their analysis is limited by their complexity.

This study aims to i) demonstrate the utility of the stress entropic load for evaluating the adaptation strain on the organism; ii) use dynamical systems theory for estimating core/surface body temperature in a given ambient temperature; iii) describe a mechanism which facilitates the evaluation of stability in the homeostatic equilibrium in a wider range of ambient air temperatures.

## Methods

In this article, we use dynamical modeling to describe human thermoregulation and compute SEL for each model’s possible trajectories. This approach enables us to simulate stress load during temperature changes in the human body. We propose a novel simple dynamical model describing the thermoregulation process. We perform standard equilibrium and bifurcation analysis of the dynamical model, specifically detecting a fold bifurcation^13^. A fold bifurcation may be associated with the hysteresis principle typical of living systems^14,15^. The analysis enables us to locate a homeostatic region^4^ and an allostatic verge^8,16^ in a dynamical system. A homeostatic region is a region defined by both parameters and initial conditions for state variables in which the homeostatic equilibrium is stable. An allostatic verge is a manifold at which a system enters or leaves a homeostatic region. We associate an allostatic verge with the hysteresis principle or, more generally, with catastrophe theory^14,15^.

In this article, we cannot use the universal Golubitsky’s and Stewart’s theory^17^ to analyze homeostatic equilibria in a dynamical system as a departure point because, in this theory, homeostasis is simplified. Instead of a process during which “some output variable remains approximately constant as an input parameter varies”^17^, it presumes that the output variable remains constant. However, such a simplification does not work outside of idealized scenarios. In reality, no physiological variable is truly constant; there is always at least a small deviation from the optimal value. Therefore, we choose not use this simplification to study the efficiency of the homeostatic mechanism.

## Results and conclusion

### Model definition

A model of mammal thermoregulation is a typical example of a homeostatic system. A simple model of thermoregulation with heating saturation was proposed by Nijhout et al. 2014^4^. Their model centers on a basic assumption, i.e. that core body temperature $T_C$ remains approximately constant in a wide range of air temperatures $T_A$^18^. Our model further develops this idea by assuming that core body temperature $T_C$ is affected by air temperature $T_A$, mostly through an additional layer of skin. Hence, both air temperature $T_A$, and core temperature $T_C$ affect skin temperature $T_S$ in our model. Skin temperature is thus a key variable for the thermodynamic modeling of entropy production^9,16^. In our model, the only direct heat transfer between the body core and the ambient air takes places through breathing. It is proportional to the volume of inhaled air, and, consequently, the volume of oxygen uptake $V_{O_2}$. The volume of oxygen uptake $V_{O_2}$ is not homeostatically regulated, meaning no known sensor in the body is capable of directly measuring it^5^. In our conceptualization of the model, we use the volume of oxygen uptake as a parameter, as i) there is no direct sensor of oxygen uptake and ii) the mechanisms involved in the regulation of oxygen uptake seem to be too complex to analyze using current methods. Our mathematical model of mammal thermoregulation may thus be presented as follows:

1 $$\begin{aligned} \frac{\text {d}T_S}{\text {d}t}&=k_1\left( T_A-T_S\right) +k_2\left( T_C-T_S\right) \end{aligned}$$

2 $$\begin{aligned} \frac{\text {d}T_C}{\text {d}t}&=k_3(T_S-T_C)+f_S\left( T_C\right) +k_4 V_{O_2} (T_A - T_C), \end{aligned}$$

where Tables 1, 2 denote time-dependent state variables and parameters, and $f_s\left( T_C\right)$ is a saturating function. Generally, when no heat loss occurs during the heat transfer between body core and skin, rate $k_2$ is equal to rate $k_3$.

**Table 1:** State variables for model (1), (2).

State variable	Description
$T_C$	Core body temperature $\left[ {\mathrm K} \right]$
$T_S$	Skin temperature $\left[ {\mathrm K} \right]$

**Table 2:** Parameters for model (1), (2).

Parameter	Description	Default value
$k_1$	Rate of heat transfer between skin and ambient air $\left[ {\mathrm{min}}^{-1} \right]$	0.2
$k_2$	Rate of heat transfer between skin and core $\left[ {\mathrm{min}}^{-1} \right]$	0.2
$k_3$	Rate of heat transfer between core and skin $\left[ {\mathrm{min}}^{-1} \right]$	0.2
$k_4$	Rate of heat transfer between core and ambient air through breathing $\left[ {\mathrm{l}}^{-1} \right]$	0.06
$T_A$	Ambient air temperature $\left[ {\mathrm K} \right]$	300
$V_{O_2}$	Oxygen consumption in $\left[ {\mathrm{l}} \, \hbox {min}^{-1} \right]$	0.3

Heat transfer rates $k_1$, $k_4 V_{O_2}$ describe the interaction of the body and environment through skin temperature $T_S$ and core body temperature $T_C$. Similarly, heat transfer rates $k_2$, $k_3$ describe the interaction between core body and skin temperature. We assume that the rate $k_4 V_{O_2}$ is significantly lower than the other rates, i.e. $k_4 V_{O_2} \ll k_1$, $k_2$, $k_3$. Saturating function $f_S$ describes the inner mechanism of the body thermoregulation regardless of the external environment. The term saturating function was introduced by Nijhout et al. 2014^4^. Heat transfer rates are illustrative and their estimation constitutes a task for future studies.

In general, saturating function $f_S$ should have the following properties:

1. $f_S$ is smooth in $T_C$.
2. There is no saturation at core temperature set point $T_C^{Set}$: $f_S\left( T_C^{Set}\right) =0$.
3. When the core temperature is lower than set point $T_C^{Set}$, saturation increases the core temperature: $T_C<T_C^{Set} \Rightarrow f_S\left( T_C\right) >0$; when the core temperature is higher than set point $T_C^{Set}$, saturation reduces the core temperature: $T_C>T_C^{Set} \Rightarrow f_S\left( T_C\right) <0$.
4. The saturation function is almost zero for a core temperature far away from the set point: $f_S\left( T_C\right) \rightarrow 0$ as $T_C\rightarrow \pm \infty$. Moreover, core temperature thresholds $T_C^{\theta _1}$, $T_C^{\theta _2}$ exist, so that if $T_C<T_C^{\theta _1}$ or $T_C>T_C^{\theta _2}$, then $f_S\left( T_C\right) \approx 0$.
5. Function $f_S$ has only one maximum for $T_C=T_C^{MAX}$, $T_C^{\theta _1}<T_C^{MAX}<T_C^{Set}$; and one minimum for $T_C=T_C^{MIN}$, $T_C^{\theta _2}>T_C^{MIN}>T_C^{Set}$.

An example of saturating function $f_s$ follows:

3 $$\begin{aligned} f_S\left( T_C\right) =p\left( T_C^{Set}-T_C\right) e^{-v\left( T_C^{Set}-T_C\right) ^2}. \end{aligned}$$

The proposed saturating function is similar to functions used in^4^; for computational reasons, we proposed a different formula to ensure $f_s$ is sufficiently smooth. The mechanism described by $f_S$ allows the body to bring the temperature back to a set-point $T_C^{Set}$^4^. The proposed saturating function is symmetric with respect to $T_C^{Set}$, but we do not require any symmetries in general. Parameters of function $f_S$ are denoted by Table 3.

**Table 3:** Parameters for saturating function $f_s$ (3).

Parameter	Description	Default value
*p*	Shape (peak) of saturating function $f_S$ $\left[ {\mathrm {min}}^{-1} \right]$	2
*v*	Shape (reciprocal of variability) of saturating function $f_S$ $\left[ K^{-2} \right]$	0.5
$T_C^{Set}$	Core body temperature set-point [*K*]	310.15

To compare saturating functions $f_S$ of different shapes, we define the area below the saturation curve $E_S \left[ {\mathrm K}^{2} min^{-1} \right]$ as

$$\begin{aligned} E_S=\int _{T_C^{\theta _1}}^{T_C^{\theta _2}} |f_S\left( T_C \right) | {\text {d}}T_C. \end{aligned}$$

**Figure 1:**
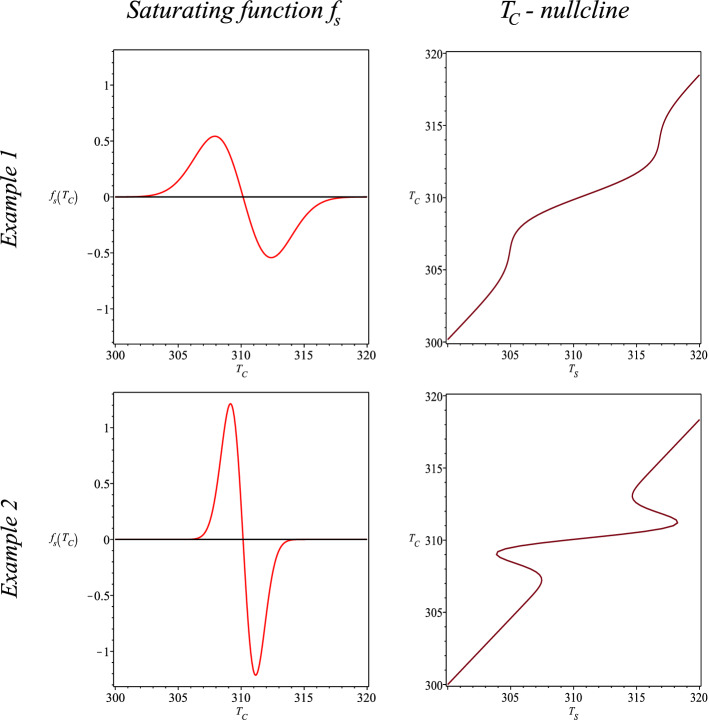
Different saturating functions with area below curve $E_S=4\; {\mathrm K}^{2} min^{-1}$ for parameters $v=0.1$, $p=0.4$ (*Example 1*) and $v=0.5$, $p=2$ (*Example 2*).

Therefore, the saturation functions of different shapes with the same area below the saturation curve exist. Figure 1 shows two different saturation functions with the same area below the saturation curve ($E_S=4\; {\mathrm K}^{2} min^{-1}$). Example 1 shows a saturation curve of a higher variability ($v=0.1$) and smaller peak ($p=0.4$) in comparison to Example 2 ($v=0.5$, $p=2$). Example 2 enables core temperature $T_C$ to stabilize closer to the set point $T_C^{Set}$ than Example 1.

We can reduce the number of parameters of model (1), (2) using the following transformation $\tau =k_1 t$, $a=\frac{k_2}{k_1}$, $b=\frac{k_3}{k_1}$, $c=\frac{p}{k_1}$, $d=\frac{k_4 V_{O_2}}{k_1}$:

$$\begin{aligned} \frac{\text {d}T_S}{\text {d}\tau }&=\left( T_A-T_S\right) +a\left( T_C-T_S\right) \\ \frac{\text {d}T_C}{\text {d}\tau }&=b(T_S-T_C)+c\left( T_C^{Set}-T_C\right) e^{-v\left( T_C^{Set}-T_C\right) ^2}+d(T_A - T_C). \end{aligned}$$

The total number of parameters is thus reduced from nine to seven. In section Model analysis, the results are presented using the original parameters, i.e. before the transformation. The transformation aims to ensure that we only study relevant parameter dependencies.

### Model analysis

The formal mathematical analysis of the model follows.

First, we analyze the equilibria of system (1), (2) for general sufficiently smooth function $f_S$. By setting the right-hand sides of equations (1), (2) equal to zero and by then simplifying the resulting two equations, we obtain an equation for core body temperature equilibria $T_C^*$.

4 $$\begin{aligned} T_A=T_C^*-\frac{k_1+k_2}{k_1 k_3+k_4 V_{O_2}\left( k_1+k_2\right) }f_S\left( T_C^* \right) . \end{aligned}$$

The stability of solutions can be determined using the Jacobi matrix *J* of system (1), (2). Using stability conditions ${\text {det }} J > 0$ and ${\text {trace }} J < 0$, we obtain stability condition

5 $$\begin{aligned} \frac{\text {d}f_S \left( T_C\right) }{\text {d}T_C}\bigg |_{T_C=T_C^*}< \frac{k_1 k_3}{k_1+k_2}+k_4 V_{O_2}. \end{aligned}$$

For arbitrary $T_A$, equation (4) has at least one solution with respect to $T_C^*$. Moreover, if $T_C^0$ exists so that

6 $$\begin{aligned} \frac{\text {d}f_S \left( T_C\right) }{\text {d}T_C}\bigg |_{T_C=T_C^0} > \frac{k_1 k_3}{k_1+k_2}+k_4 V_{O_2}, \end{aligned}$$

it is then possible to establish $T_A$ for which equation (4) has three solutions with respect to $T_C^*$. The solutions are either

- solution 1: $T_C^* < T_{{\text {inf}}_1}$, solution 2: $T_{{\text {inf}}_1}<T_C^*<T_{{\text {sup}}_1}$, and solution 3: $T_{{\text {sup}}_1}<T_C^*<T_C^{Set}$; for $T_{{\text {inf}}_1} = {\text {inf}}_{\mathbb {R}} \left\{ T_C^0: T_C^0<T_C^{Set} {\text { and condition }}(6) \hbox { holds} \right\}$, and $T_{{\text {sup}}_1} = {\text {sup}}_{\mathbb {R}} \left\{ T_C^0: T_C^0<T_C^{Set} {\text { and condition }} (6) \hbox { holds} \right\}$ or
- solution 1: $T_C^* > T_{{\text {sup}}_2}$, solution 2: $T_{{\text {sup}}_2}>T_C^*>T_{{\text {inf}}_2}$, and solution 3: $T_{{\text {inf}}_2}>T_C^*>T_C^{Set}$; for $T_{{\text {inf}}_2} = {\text {inf}}_{\mathbb {R}} \left\{ T_C^0: T_C^0>T_C^{Set} {\text { and condition }}(6) \hbox { holds} \right\}$, and $T_{{\text {sup}}_2} = {\text {sup}}_{\mathbb {R}} \left\{ T_C^0: T_C^0>T_C^{Set} {\text { and condition }}(6) \hbox { holds} \right\}$.

The condition (5) indicates that solutions 1 and 3 are stable. Depending on $T_A$, typically, one of the following critical cases occurs: either solutions 1 and 2 collide, forming a saddle-node equilibrium $T_C^*=T_{{\text {inf}}_1}$, or $T_C^*=T_{{\text {sup}}_2}$, or solutions 2 and 3 collide, forming a saddle-node equilibrium $T_C^* = T_{{\text {sup}}_1}$, or $T_C^* = T_{{\text {inf}}_2}$.

The following paragraphs describe the consequences of this theorem, and present the results for $f_S$ as defined by equation (3). We analyzed system (1), (2), (3) with parameters from Tables 2 and 3 using equilibria and bifurcation detection and continuation methods in Matcont software^19,20^, and using the DETools package in Maple software^21^. The goal of the analysis is to locate allostatic verges depending on parameters from 2 and 3. In different settings, allostatic verges can also depend on other quantities.

**Figure 2:**
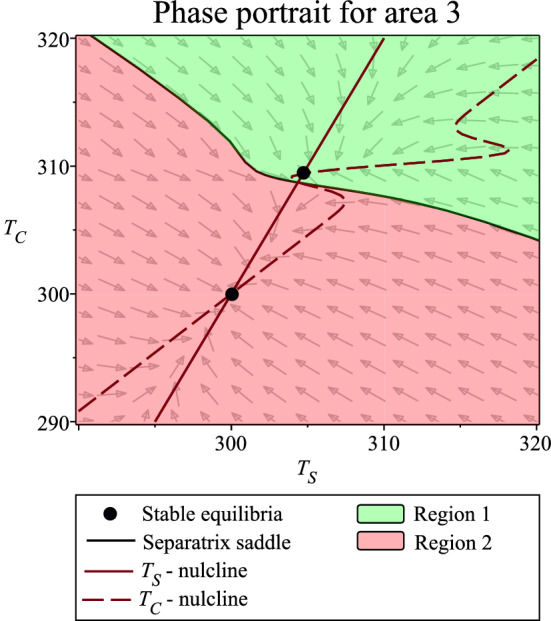
Phase portrait for area 3 for parameter values given by Tables 2 and 3.

Depending on system parameters, two possible stable equilibria of system (1), (2), (3) exist. We can distinguish between them using core temperature $T_C$ at the equilibrium point. Core temperature $T_C$ at the first equilibrium point is close to the core temperature set-point $T_C^{Set}$. We thus call the equilibrium homeostatic. Core temperature $T_C$ at the second equilibrium point is close to ambient air temperature $T_A$. We call this equilibrium non-homeostatic. We divided the entire parameter space into areas where either a homeostatic equilibrium exists and is stable (area 1), where a non-homeostatic equilibrium exists and is stable (area 2), or where both equilibria exist and are stable (area 3). By setting parameters to values from area 3, one additional saddle equilibrium exists, see Fig. 2. The separatrices of the saddle divide phase space, i.e. the space of state variables $T_C$ and $T_S$, into two regions (region 1, region 2). Region 1 is a basin of attraction of the homeostatic equilibrium. Region 2 is a basin of attraction of the non-homeostatic equilibrium.

The existence of bi-stability in area 3 is closely connected to a one-parameter dependent hysteresis phenomenon. Since in this model, hysteresis is a mechanism through which our system leaves or enters a homeostatic region, we analyze hysteresis to study allostatic verges. We identified the hysteresis phenomenon for air temperatures $T_A$ smaller than the core temperature set point $T_C^{Set}$. Similarly, it can be described for air temperatures $T_A$ larger than the core temperature set point $T_C^{Set}$. To identify the hysteresis phenomenon from either the trajectories of the dynamical system or the measured time series, we focus on two model scenarios of parameter change: (i) increasing and (ii) decreasing ambient air temperature $T_A$. In the first scenario we start at a stable non-homeostatic equilibrium for $T_A$ below 307 K. We slowly increase $T_A$. System (1), (2), (3) stabilizes on a non-homeostatic equilibrium until $T_A$ reaches an allostatic verge of 307 K. After crossing this allostatic verge, system (1), (2), (3) subsequently stabilizes on a homeostatic equilibrium until $T_A$ reaches the next allostatic verge at 321 K. In the second scenario we start at a homeostatic equilibrium for $T_A$ between 299 K and 321 K. We slowly decrease $T_A$. System (1), (2), (3) stabilize on a homeostatic equilibrium until $T_A$ reaches an allostatic verge of 299 K. After crossing this allostatic verge, system (1), (2), (3) subsequently stabilizes on a non-homeostatic equilibrium. Allostatic verges are different for increasing and decreasing ambient air temperature scenarios (307 K and 299 K).

Moreover, allostatic verges are also dependent on other parameters of system (1), (2), (3). To describe the dependencies of allostatic verges on parameters, we studied several two-parameter sections of the parameter space. We provide the analysis in the following paragraphs.

**Figure 3:**
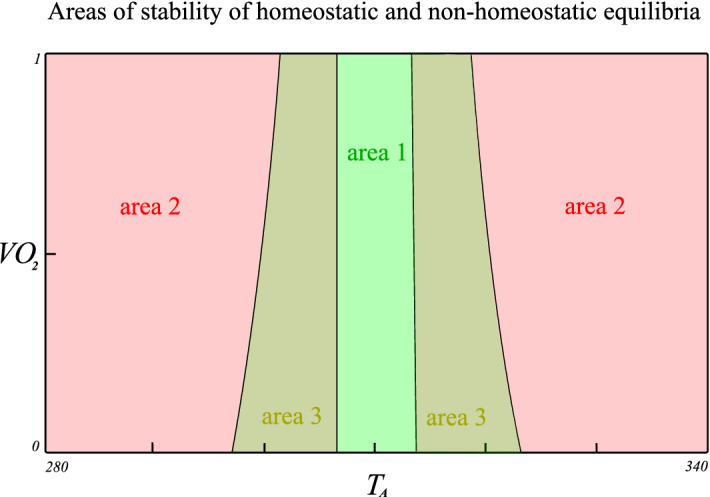
Transversal section through areas of stability of equilibria for parameter values given by Tables 2 and 3 and free parameters $T_A$ and $V_{O_2}$.

We studied the equilibria of system (1), (2), (3) depending on ambient air temperature $T_A$ and oxygen consumption $V_{O_2}$. For smaller $V_{O_2}$ area 3 gets wider, the change does not affect the size of area 1, see Fig. 3 and Table 4.

**Table 4:** Estimated allostatic verges of ambient air temperature $T_A [{\mathrm K}]$ on the borders of areas 1, 2 and 3 for selected values of $V_{O_2}$.

	Allostatic verges of $T_A \left[ {\mathrm K}\right]$ on borders of areas			
$V_{O_2}$	Area 2–3	Area 3–1	Area 1–3	Area 3–2
0.1	298	307	313.5	322.5
0.4	299.5	307	313.5	320.5
0.7	301	307	313.5	319.5

In addition to $T_A$ and $V_{O_2}$, the areas of stability differ depending on the shape of saturation function $f_S$. We study the dependence of ambient air temperature $T_A$ and the shape of $f_S$. For the same area below the saturation curve, the higher peak *p* and the smaller variability $\frac{1}{v}$ enlarge area 3 and narrow area 1. Table 5 presents an example of such a dependency with the area below the saturation curve $E_S=4 {\mathrm K}^2 min^{-1}$.

**Table 5:** Estimated allostatic verges of the ambient air temperature $T_A [{\mathrm K}]$ on borders of areas 1, 2 and 3 depending on the shape of the saturation curve (described by some values of parameters *p*, *v*) for the area below the saturation curve $E_S=4 {\mathrm K}^2 min^{-1}$.

Shape		Allostatic verges of $T_A [{\mathrm K}]$ on borders of areas			
*v*	*p*	Area 2–3	Area 3–1	Area 1–3	Area 3–2
1	4	295	307.5	312.5	325
0.9	3.6	296	307.5	312.5	324.5
0.8	3.2	296.5	307.5	313	323.5
0.7	2.8	297.5	307.5	313	323
0.6	2.4	298	307	313	322
0.5	2	299	307	313.5	321
0.4	1.6	300	306.5	314	320
0.3	1.2	301	306	314	319
0.2	0.8	302	305.5	315	318
0.1	0.4	303.5	304	316.5	317

The following Table 6 summarizes obtained results in terms of the homeostatic region. The necessity to characterize this homeostatic region by state-variables comes from the bi-stability between two possible equilibria for parameter area 3. The bi-stable mechanism enables the body to remain in a homeostatic equilibrium for more extreme temperatures during air temperature increase or decrease. On the other hand, the mechanism causes the destabilization of homeostatic equilibrium through even small perturbations.

**Table 6:** Homeostatic regions for mammal thermoregulation model (1) and (2).

	Parameters	State-variables
Homeostatic region 1	Area 1	Arbitrary $T_C$, $T_S$
Homeostatic region 2	Area 3	Region 1

For areas see Fig. 3 and Tables 4 and 5. For regions see Fig. 2.

Finally, having studied a dynamical system of mammal thermoregulation (1), (2), (3), we proceed to link our results to the stress entropic load concept. Assuming we are studying a short term event, we can use system (1), (2), (3) and the simplified formula published by Zlámal et al. 2018^9^ to compute the immediate change of the stress entropic load $\Delta s_{\text {SEL}}$; see Supporting information for the formula. Our model describes a dynamic of core temperature $T_C$ and skin temperature $T_S$ depending on several parameters, including ambient air temperature $T_A$ and oxygen consumption $V_{O_2}$. In the $\Delta s_{\text {SEL}}$ formula, the only free variable left is the CO$_{2}$ liberation rate. As CO$_{2}$ is the only free variable in the formula, the human body can thus optimize its stress entropic load by changing the CO$_{2}$ liberation rate. The mechanism of regulating CO$_{2}$ can therefore ensure that the following holds true in the homeostatic equilibrium: $\frac{\text {dSEL}\left( t\right) }{\text {d}t}=0$. Therefore, SEL production is zero at the homeostatic equilibrium, which is in perfect agreement with the underlying theory that SEL is directly related to stress levels recorded at a given point in time.

## Discussion

In this paper, we propose a novel model of human thermoregulation using the concept of stress entropic load as a departure point. In doing so, we propose changes to the usual model of human thermoregulation in order to i) include all necessary variables for SEL computation into the model, such as core temperature $T_C$, skin temperature $T_S$, ambient temperature $T_A$, oxygen consumption $V_{O_2}$, and ii) model different adaptation strategies. Our results suggest that humans may use two types of adaptation strategies with respect to thermoregulation. The first adaptation strategy type takes into account adaptation costs measured by the area below the saturation curve $f_S$ while the second is based on CO$_{2}$ production.

As for the first strategy: We studied the bi-stable area in system (1), (2) depending on the shape of the saturating function $f_S$ and oxygen consumption $V_{O_2}$. The bi-stable mechanism enables the body to remain in a homeostatic equilibrium state for more extreme temperatures, but it leads to the possible destabilization of the homeostatic equilibrium through even small perturbations. With the decrease of oxygen consumption $V_{O_2}$, the area of bi-stability (area 3) increases, but the homeostatic equilibrium area of stability remains approximately the same, see Fig. 3. Therefore, as oxygen consumption decreases, body can remain in homeostasis for more extreme temperatures. Similarly, depending on the shape of saturation sizes of both area 1 and area 3 changes. For the same area below the saturation curve, the saturating function with high peaks and small variability enlarges area 3 and narrows area 1; see Table 5. Therefore, body can remain in the homeostatic equilibrium for more extreme temperatures; however, the local stability is more fragile due to the limited resources needed to maintain homeostasis.

The thermoregulation mechanism described in our paper differs from classical chair-like relationships^4,17^. Golubitsky and Stewart^17^ assumed that the derivative of the variable exhibiting homeostasis with respect to the parameter vanishes at homeostatic equilibrium. For the chair-like relationship, they assumed two additional conditions^17^. In our example of system (1), (2) with (3) the derivative of core temperature with respect to ambient temperature $T_C^*\left( T_A\right)$ does not vanish for any saturating function shape (defined by parameters *p* and *v*). However, the implicit derivative of $T_C^*\left( T_A\right)$ in the homeostatic equilibrium tends to zero as *p* tends to infinity. The theory is therefore not sufficient to describe this type of homeostatic system.

As for the second adaptation strategy which builds on CO$_{2}$ production, we can use the dynamical systems of mammal thermoregulation to compute the change in stress entropic load (SEL) for short-term events ^9^. Interestingly, our results show that the body can attain zero change in SEL at homeostatic equilibrium by manipulating the CO$_{2}$ liberation rate. This is supportive of the notion that changes in SEL are representative of the current adaptation costs of the organism. In the light of our results, it is essential to mention that respiratory control in humans is primarily maintained via the partial pressure of CO$_{2}$ in peripheral blood. The complete adaptation process consists of balancing both costs of adaptation, represented in the model by saturating function $f_S$, and stress entropic load (SEL) through the CO$_{2}$ liberation rate. Our findings may thus be interpreted to signify that manipulation with CO$_{2}$ levels could reduce adaptation costs for thermoregulation, which may result in an increase of energy reserves for other purposes such as healing and regeneration.

## Supplementary Information

Supplementary Information.
